# Dataset on functional and chemical properties of the medicinal halophyte *Polygonum maritimum* L. under greenhouse cultivation

**DOI:** 10.1016/j.dib.2019.104357

**Published:** 2019-08-19

**Authors:** Maria João Rodrigues, Ivo Monteiro, Chloé Placines, Viana Castañeda-Loaiza, Sylwester Ślusarczyk, Adam Matkowski, Catarina Pereira, Pedro Pousão-Ferreira, Luísa Custódio

**Affiliations:** a Centre of Marine Sciences, University of Algarve, Faculty of Sciences and Technology, Ed. 7, Campus of Gambelas, 8005-139, Faro, Portugal; b IPMA, Aquaculture Research Station, Olhão, Portugal; c Department of Pharmaceutical Biology and Botany, Wroclaw Medical University, Wroclaw, Poland

**Keywords:** Antioxidant activity, Anti-inflammatory activity, Halophyte domestication, Saline agriculture

## Abstract

This data article includes data and analyses on the effect of different agronomic techniques on the production of *Polygonum maritimu*m L. (sea knotgrass), namely different salinity irrigation treatments (0, 100, 200, 300 and 600 mM of NaCl) and a multi-harvest regime, and their relation with the chemical profile (ultra-high-resolution mass spectrometry - UHRMS), *in vitro* antioxidant [radical-scavenging activity (RSA) of DPPH and ABTS, copper chelating activity and ferric reducing antioxidant power] and anti-inflammatory (nitric oxide reduction on lipopolysaccharide-stimulated macrophages) activities. For further interpretation of the data presented in this work, please see the related research article “The irrigation salinity and harvesting affect the growth, chemical profile and biological activities of *Polygonum maritimum* L.” (Rodrigues et al., 2019).

**Table:** Specifications Table

Subject area	Agronomy, biology, chemistry
More specific subject area	Biological and chemical profiling of *Polygonum maritimum* cultivated under saline irrigation
Type of data	Table, graph, figure
How data was acquired	Photospectrometer (Biotek synergy 4), liquid chromatography (LC) − electrospray ionization (ESI)-QTOF-MS) (Thermo Dionex Ultimate 3000 RS).
Data format	Raw and analyzed
Experimental factors	12-week plants were subjected to different salinity irrigation treatments (freshwater, 100, 200, 300 and 600 mM of NaCl), followed by a multi harvesting regime with a 6-week interval.
Experimental features	Plants from different conditions were freeze-dried and resultant biomass extracted with acetone (1:40, w/v) and analyzed for their *in vitro* antioxidant and anti-inflammatory properties and chemical profile by LC-UHRMS.
Data source location	Centre of Marine Sciences, University of Algarve, Faculty of Sciences and Technology, Ed. 7, Campus of Gambelas, 8005-139 Faro, Portugal.
Data accessibility	Data provided within this article.
Related research article	M.J. Rodrigues, I. Monteiro, C. Placines, V. Castañeda-Loaiza, S. Ślusarczyk, A. Matkowski, C. Pereira, P. Pousão-Ferreira, L. Custódio, The irrigation salinity and harvesting affect the growth, chemical profile and biological activities of *Polygonum maritimum* L., Ind. Crop. Prod. 139 (2019) 111 510. https://doi.org/10.1016/j.indcrop.2019.111510[1].

**Table:** 

**Value of the data** • The first dataset on the effect of agronomic techniques (irrigation salinity and harvesting) on the chemical profile and *in vitro* antioxidant and anti-inflammatory properties of the medicinal halophyte *P. maritimum* (sea knotgrass) • This dataset provides relevant information to other researchers for understanding the influence of cultivation conditions, including saline irrigation and multi-harvest regime, on halophyte plants functional properties • Data could be relevant for the improvement of sustainable production of halophytes using salinized soils or brackish waters, as high value-added crops for commercial purposes.

## 1 Data

**Fig. 1:**
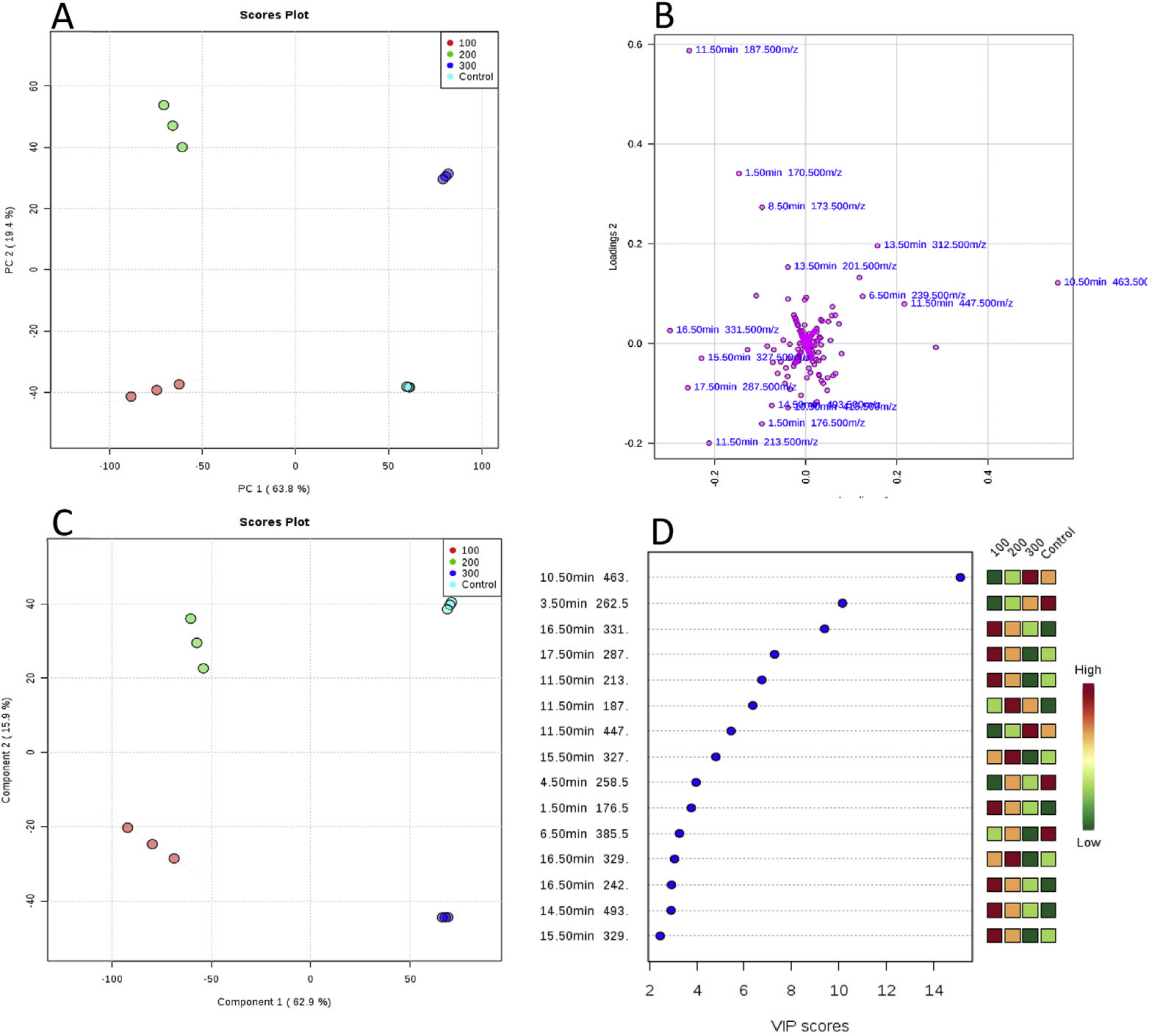
**(A)** PCA scores plot based on UHPLC−MS data showing separation amongst samples from different irrigation conditions (0 mM - Light Blue; 100 mM – Red; 200 mM – Green; and 300 mM - Deep Blue) for the 1st harvest, together with their respective 95% confidence regions. The explained variances are shown in brackets (PC 1 and PC 2: 63.8 and 19.4%, respectively). **(B)** The corresponding loadings scatter plot showing the compounds (represented by their retention times [RT]) that are correlated to separation in scores plot. **(C)** PLS-DA of metabolites between groups described above (component 1 and component 2: 62.9 and 15.9%, respectively)**. (D)** Variables important in projection (VIP) scores of 15 top contributors (shown as RT) to PLS-DA component 1.

**Fig. 2:**
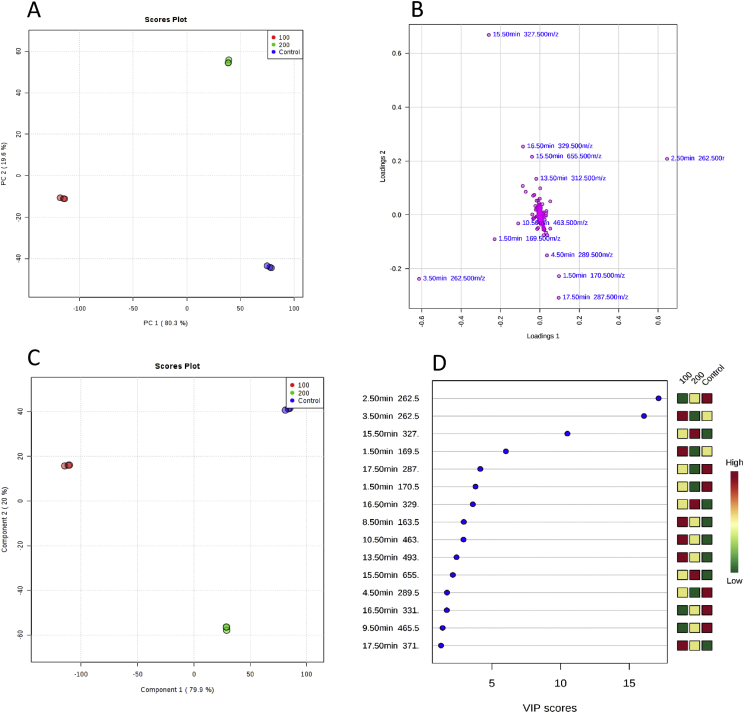
**(A)** PCA scores plot based on UHPLC−MS data showing separation amongst samples from different irrigation conditions (0 mM - Deep Blue; 100 mM – Red; and 200 mM – Green) for the 2nd harvest, together with their respective 95% confidence regions. The explained variances are respectively 80.3 and 19.6% for PC 1 and PC 2. **(B)** The corresponding loadings scatter plot showing the compounds (shown as RT) that are correlated to separation in scores plot. **(C)** PLS-DA of metabolites between groups described above (component 1 and component 2 are 79.9 and 20%, respectively)**. (D)** Variables important in projection (VIP) scores of 15 top contributors (presented as RT) to PLS-DA component 1.

**Fig. 3:**
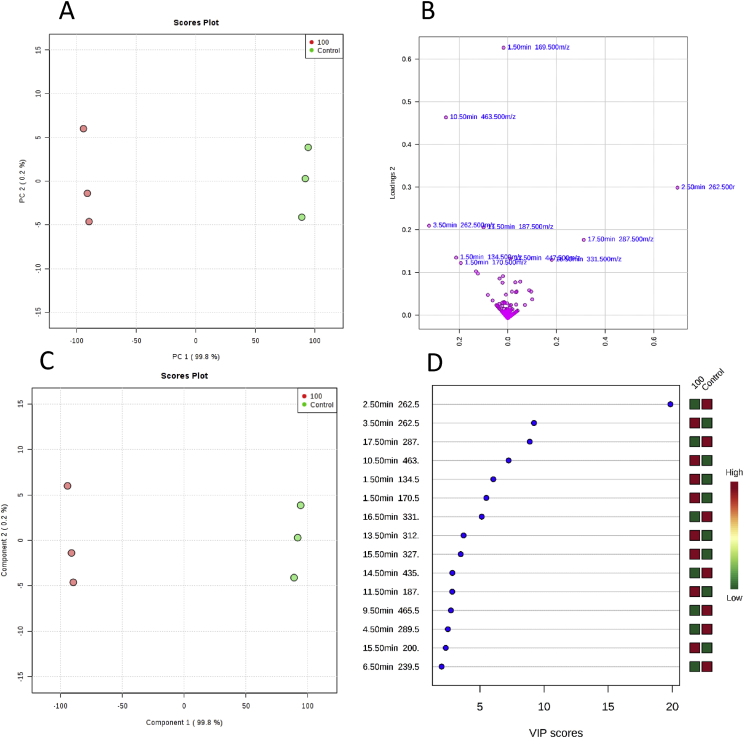
**(A)** PCA scores plot based on UHPLC−MS data showing separation samples from different irrigation conditions (0 mM - Green; and 100 mM – Red) for the 3rd harvest, together with their respective 95% confidence regions. The explained variances are 99.8 and 0.2% for PC 1 and PC 2, correspondingly. **(B)** The corresponding loadings scatter plot showing the compounds (represented by their RT) that are correlated to separation in scores plot. **(C)** PLS-DA of metabolites between groups described above (component 1 and component 2: 99.8 and 0.2%, respectively)**. (D)** Variables important in projection (VIP) scores of 15 top contributors (displayed as RT) to PLS-DA component 1.

The sea knotgrass plants were produced in a greenhouse under different irrigation conditions (freshwater, 100, 200 and 300 mM of NaCl), and submitted to three consecutive harvests. Obtained biomass (above ground organs) were extracted with acetone, and the extracts were tested for *in vitro* antioxidant [radical-scavenging activity (RSA) of DPPH and ABTS, copper chelating activity (CCA) and ferric reducing antioxidant power (FRAP)] and anti-inflammatory (nitric oxide reduction on lipopolysaccharide-stimulated macrophages) properties. The results of half maximal inhibitory concentration (IC_50_) are reported in Table 1, Table 2, for antioxidant and anti-inflammatory, respectively. For the same treatment, the RSA towards DPPH and ABTS, and CCA increased with the harvest. The same tendency was observed in FRAP, except on freshwater-irrigated plants that showed decreased activity from 1st to 2nd harvest, however decreasing in the 3rd harvest. The anti-inflammatory activity decreased with the harvest, and the lowest IC_50_ values were obtained on biomass from the 1st harvest, for all treatments. A detailed chemical profiling was performed by LC-UHRMS [1] and differences between treatments and harvests were analysed by PCA and PLC-DA statistics (Fig. 1, Fig. 2, Fig. 3). Striking differences on the chemical composition of statistically significant peaks tends to differ along with consecutive harvests and showed clear separation of salt concentration treatments disregarding the harvest sequence.

**Table 1:** *In vitro* antioxidant activities of the acetone extract of sea knotgrass aerial parts irrigated with freshwater (approximately 0 mM of NaCl) and artificial saltwater with different NaCl concentrations (100, 200 and 300 mM of NaCl), for 3 repeated harvests. Results are expressed as IC_50_ values (μg/mL).

Assay	Harvest	0 mM NaCl	100 mM NaCl	200 mM NaCl	300 mM NaCl
DPPH	1st	nd	nd	nd	679 ± 13^c^
	2nd	nd	nd	584 ± 29^b^	–
	3rd	138 ± 2^a^	664 ± 12^c^	–	–
	BHT^∗^	111 ± 1^a^			
ABTS	1st	nd	nd	nd	705 ± 30^c^
	2nd	704 ± 10^c^	728 ± 37^c^	nd	–
	3rd	279 ± 27^b^	nd	–	–
	BHT^∗^	140 ± 1^a^			
CCA	1st	nd	nd	nd	nd
	2nd	nd	nd	nd	–
	3rd	560 ± 22^b^	nd	–	–
	EDTA^∗^	171 ± 9^a^			
FRAP	1st	182 ± 5^cd^	389 ± 15^f^	216 ± 9^de^	273 ± 17^e^
	2nd	277 ± 10^e^	233 ± 11^de^	145 ± 6^bc^	–
	3rd	54 ± 4^a^	117 ± 8^b^	–	–

-: Samples that did not survived until the harvest; nd: not determined (activity lower than 50% at 1000 μg/mL); ^∗^: positive control. Values represent the mean ± standard error of the mean (SEM) of four experiments (n = 4). For the same assay, values followed by different letters (DPPH and ABTS: a–c; CCA: a–b; and FRAP: a–f) are significantly different at *P* < 0.05 (Tukey HSD test).

**Table 2:** *In vitro* anti-inflammatory activity of the acetone extract of *P. maritimum* aerial parts irrigated with freshwater (approximately 0 mM of NaCl) and artificial saltwater with different NaCl concentrations (100, 200 and 300 mM of NaCl). Results are expressed as IC_50_ values (μg/mL).

Harvest	0 mM NaCl	100 mM NaCl	200 mM NaCl	300 mM NaCl
1st	53.1 ± 2.1^b^	52.8 ± 3.4^b^	53.7 ± 2.0^b^	51.4 ± 8.7^b^
2nd	nd	nd	42.7 ± 5.5^ab^	–
3rd	87.7 ± 1.6^c^	nd	–	–
L-NAME^∗^	27.6 ± 2.2^a^			

-: Samples that did not survived until the harvest; nd: not determined (activity lower than 50%); ^∗^: positive control. Values represent the mean ± standard error of the mean (SEM) of four experiments (n = 4). Values followed by different letters (a–c) are significantly different at *P* < 0.05 (Tukey HSD test).

## 2 Experimental design, materials, and methods

### 2.1 Extraction

The dried biomass of aerial parts (leaves, stems and shoots) was extracted with pure acetone (1:40, w/v) in an ultrasonic bath [1]. The extracts were filtered (Whatman no. 4) and acetone was removed by rotary evaporation. The dried extracts were weighed, resuspended in methanol at 10 mg/mL, and stored at −20 °C.

### 2.2 Radical-scavenging activity (RSA) on DPPH and ABTS

The DPPH and ABTS RSA of the extracts at different concentrations (10–1000 μg/mL) was performed as reported earlier [2]. Differences in absorbance were measured in a microplate reader (Biotek Synergy 4). Butylated hydroxytoluene (BHT) was used as standard at concentrations equal to those of the samples. Results were expressed as an inhibition percentage, comparative to a control containing methanol instead of the sample, and as half maximal inhibitory concentration (IC_50_ values, μg/mL).

### 2.3 Ferric reducing antioxidant power (FRAP)

The extracts’ capacity to reduce Fe^3+^ (at concentrations amongst 10–1000 μg/mL) was evaluated as described by Rodrigues et al. [2]. An increase in the absorbance at 700 nm in the reaction mixture indicates an increased reducing power of the samples (Biotek Synergy 4). Results were calculated as a percentage in relation to the standard (BHT, 1000 μg/mL), and as IC_50_ values (μg/mL).

### 2.4 Metal chelating activity on copper (CCA)

The CCA of the extracts (at concentrations varying between 10 and 1000 μg/mL) was assayed as depicted before [2]. The color switch was measured on a microplate reader (Biotek Synergy 4), and ethylenediaminetetraacetic acid (EDTA) was applied as the positive control at the identical concentrations of the extracts. Results were presented as an inhibition percentage comparatively to a control using methanol as substitute of the sample, and as IC_50_ values (μg/mL).

### 2.5 Cell culture and cell viability

RAW 264.7 cells were grown in RPMI 1640 culture medium complemented with 10% heat-inactivated fetal bovine serum (FBS), 1% L-glutamine (2 mM), and 1% penicillin (50 U/mL)/streptomycin (50 μg/mL) and were kept at 37 °C in moistened environment with 5% CO_2_. Cells were seeded at a concentration of 1 × 10^4^ cells/well, in 96-well microplates. After 24h of incubation, the extracts were added at concentrations from 3 to 100 μg/mL, and incubated for 24h. Cells treated with the vehicle (0.5% DMSO, v/v) were used as negative control, and cell viability was assessed through the 3-(4,5-dimethylthiazol-2-yl)-2,5-diphenyltetrazolium bromide (MTT) colorimetric test [3]. Results were calculated as a percentage of cell viability, in comparison with the control cells.

### 2.6 *In vitro* anti-inflammatory assay

The samples were tested for their capacity to decrease nitric oxide (NO) production in RAW 264.7 macrophages [3]. Cells were plated at 2.5 × 10^5^ cells/well in 96-well plates and left to adhere overnight. Then, non-toxic concentrations of the extracts (>80% of cell viability) were incubated in serum- and phenol-free culture medium, with 100 ng/mL of LPS, for 24h. The cellular NO production was evaluated by the Griess method [3]. Results were expressed as a percentage (%) of NO production comparing to a control cells containing DMSO (0.5%, v/v), and as IC_50_ values (μg/mL).

### 2.7 Liquid chromatography/ultra-high-resolution mass spectrometry (LC-UHRMS)

Samples were pre-treated using solid phase extraction as follows: 100 mg of the extracts were suspended in 1 mL of 0.2% formic acid in purified water (HLP10Uv, Hydrolab, Gdańsk). Next, the suspension was loaded to the C18 Sep-Pak cartridges (1 cm^3^, 360 mg, Waters Corp., Milford, MA) and washed with 0.5% methanol to remove carbohydrates and then with 80% methanol to elute phenolics. The phenolic fraction was re-evaporated, dissolved in 1 mL of 0.2% formic acid in 80% aqueous methanol, centrifuged for 5 min at 23 000×*g*, and filtered through 0.22 μm syringe filters (mix cellulose esters, Carl Roth, Karlsruhe, Germany) before LC-MS analysis (stored at −20 °C before analysis for no longer than 3 days). All analyses were performed in triplicate for three independent samples.

Liquid chromatography (LC) − electrospray ionization (ESI)-QTOF-MS was carried out using Thermo Dionex Ultimate 3000 RS system consisting of a binary pump system, sample manager, column manager and a DAD detector (Thermo Fischer Scientific, Waltham, MA), coupled to a Bruker Compact quadrupole time-of-flight (QTOF) mass spectrometer (Bruker Daltonics, Billerica, MA). Separations were performed on a Kinetex C18 column (2.1 × 100 mm, 2.6 μm, Phenomenex, USA), with mobile phase A consisting of 0.1% (v/v) formic acid in water and mobile phase B containing 0.1% (v/v) formic acid in acetonitrile. A linear gradient from 1% to 60% phase B in phase A over 20 minutes was used to separate phenolic compounds. The flow rate was 0.4 mL/min, and the column was held at 30 °C. Mass spectra were acquired in negative-ion mode with 5 Hz frequency over a mass range from m/z 100 to 1500. Operating settings of the ESI ion source were as follows: capillary voltage 3 kV, dry gas flow 6 L/min, dry gas temperature 200 °C, nebulizer pressure 0.7 bar, collision radio frequency 700.0 V, transfer time 100.0 μs, and pre-pulse storage 7.0 μs. Ultrapure nitrogen was used as drying and nebulizer gas, and argon was used as the collision gas. The collision energy was set automatically from 15 to 75 eV depending on the m/z of the fragmented ion. For calibration of the accurate mass measurements, we used sodium formate introduced to the ion source at the beginning and end of each separation via a 20 μL loop. After data acquisition, raw UPLC−QTOF-MS spectra (negative mode) were pre-processed using a ProfileAnalysis software (version 2.1, Bruker Daltonik GmbH, Germany). Parameters of ProfileAnalysis were used as follows: advanced bucket generation with retention time range of 0–20 min, mass range of 100–800 m/z, each bucket (spectral bins) was formed with 1 min and 1 m/z delta, 0.2 kernelizing value, without normalization, background subtraction, and time alignment. LC-MS analyses were processed with the Find Molecular Futures (FMF) function to create compounds (molecular features) with S/N- 3 for peak detection. Generated bucket table consisting of tR:m/z pairs and respective compound intensity was exported and uploaded to MetaboAnalyst program. Each obtained dataset was filtered and normalized to the sum of peak areas and mean-centered scaling.

Acquired spectra were processed with Bruker DataAnalysis 4.3 software. The quality of the isotopic fit was expressed by the mSigma-value. The matched peaks from SmartFormula3D were sent to MetFrag website for computer-assisted *in silico* fragmentation and identification of metabolite mass spectra. Additionally, we searched the web-based databases for potential matches to the detected compounds: the human metabolome database (http://www.hmdb.ca/), the BiGG database (http://bigg.ucsd.edu/), the PubChem database (http://pubchem.ncbi.nlm.nih.gov/), the MassBank database (http://www.massbank.jp), KEGG (www.genome.jp) and the Metlin database (http://metlin.scripps.edu).

### 2.8 Statistical analysis

Results were expressed as the mean ± standard error of the mean (SEM) of at least three repetitions. Significant differences were evaluated by analysis of variance (ANOVA) and by the Tukey HSD test (*P* < 0.05). Statistical analyses were made using the XLSTAT statistical package for Microsoft Excel (version 2013, Microsoft Corporation). The IC_50_ values were calculated by a sigmoidal fitting of the data (GraphPad Prism v. 5.0 program).

## References

[1] Rodrigues M.J., Monteiro I., Placines C., Castañeda-Loaiza V., Ślusarczyk S., Matkowski A., Pereira C., Pousão-Ferreira P., Custódio L. (2019). The irrigation salinity and harvesting affect the growth, chemical profile and biological activities of *Polygonum maritimum* L. Ind. Crops Prod..

[2] Rodrigues M.J., Soszynski A., Martins A., Rauter A.P., Neng N.R., Nogueira J.M.F., Varela J., Barreira L., Custódio L. (2015). Unravelling the antioxidant potential and the phenolic composition of different anatomical organs of the marine halophyte *Limonium algarvense*. Ind. Crops Prod..

[3] Rodrigues M.J., Gangadhar K.N., Vizetto-Duarte C., Wubshet S.G., Nyberg N.T., Barreira L., Varela J., Custódio L. (2014). Maritime halophyte species from southern Portugal as sources of bioactive molecules. Mar. Drugs.

